# Development and Validation of HPLC-DAD/FLD Methods for the Determination of Vitamins B_1_, B_2_, and B_6_ in Pharmaceutical Gummies and Gastrointestinal Fluids—In Vitro Digestion Studies in Different Nutritional Habits

**DOI:** 10.3390/molecules30193902

**Published:** 2025-09-26

**Authors:** Georgios Kamaris, Nikoletta Pantoudi, Catherine K. Markopoulou

**Affiliations:** Laboratory of Pharmaceutical Analysis, Department of Pharmacy, Aristotle University of Thessaloniki, 54124 Thessaloniki, Greece; kamarisg@pharm.auth.gr (G.K.); npantou@pharm.auth.gr (N.P.)

**Keywords:** thiamine (vitamin B_1_), riboflavin (vitamin B_2_), pyridoxine (vitamin B_6_), thiochrome, pharmaceutical gummies, HPLC-DAD/FLD, pre-column derivatization, solid phase extraction (SPE), in vitro digestion study

## Abstract

Two HPLC-based analytical methods, one with DAD and the other with an FLD detector, were developed and validated for the simultaneous analysis of B_1_, B_2_, and B_6_ vitamins, both in pharmaceutical gummies and in gastric and intestinal fluids (with water or milk or orange juice). For the detection of B_1_ by fluorometry, a pre-column oxidation/derivatization process was accomplished in the presence of B_2_ and B_6_ vitamins. The methods were performed on an Aqua column (250 mm × 4.6 mm, 5 mm) at 40 °C, with isocratic elution (70% NaH_2_PO_4_ buffer pH 4.95 and 30% methanol) and a flow rate of 0.9 mL/min. Both were validated according to ICH specifications in terms of linearity (R^2^ > 0.999), accuracy (% Mean Recovery 100 ± 3%) and precision (%RSD < 3.23). For the analysis of the samples, a stability study (in diluents, pH and fluids) was conducted, while for their purification two different extraction procedures, a liquid/solid for the gummies (%Recovery > 99.8%) and a Solid Phase Extraction (SPE) for the Gastrointestinal (G.I.) fluids, (%Recovery 100 ± 5%) were developed. Finally, to investigate whether the co-administration of B-complex with water, orange juice or milk plays a significant role in their release from gummies, a three-phase in vitro digestion protocol was applied. The results did not show significant differences with a slight superiority in the release of B_2_ and B_6_ with water, while B_1_ with orange juice.

## 1. Introduction

Vitamins are nutrients that our bodies need, in small amounts, to stay healthy. They are considered essential because most of them cannot be produced by humans but are required for survival. Unfortunately, due to modern dietary habits, the current trend is to consume insufficient amounts of certain vitamins, which raises serious public health concerns [1,2,3].

One of the main reasons is the specific dietary choices (low-carbohydrate) of some people, for burning fat and controlling their weight, which, however, do not always meet the body’s needs. Typical examples are the ketogenic, carnivore, and Atkins diets, which, due to the restriction of carbohydrates, significantly reduce the intake of foods rich in vitamins B_1_ (thiamine), B_2_ (riboflavin), and B_6_ (pyridoxine) while at the same time, due to the high consumption of meat, increase the intake of B_12_ [4,5,6,7].

However, the benefits of vitamins B_1_, B_2,_ and B_6_ in the human body are countless and deserve special attention. As for B_1_, in addition to its undeniable beneficial effect on energy production and the smoother functioning of the nervous system and brain [8,9], there is strong evidence that it improves cardiovascular function risk [10]. Particularly for the female population, its actions are associated with urogenital and reproductive health, hormonal fluctuations during the menstrual cycle, pregnancy, and breastfeeding [11]. Similarly, B_2_ participates both in energy metabolism, activating primary metabolic pathways, and in the homeostasis of the individual’s overall energy balance [12]. Finally, the need for adequate intake of vitamin B_6_ is evident, as it is related to the smooth functioning of the brain and Central Nervous System (CNS) [13] in terms of sleep induction [14] and the control of anxiety and depression [15]. Furthermore, B_6_ appears to be related to the immune system, compensating for the lack of response of pyridoxal 5′-phosphate in plasma [13].

Therefore, since insufficient intake of these vitamins from the individual’s diet over long periods of time causes fatigue, inability to concentrate, and mood disorders, their administration by nutritional supplements is considered necessary. A variety of vitamin B complex preparations are available on the market (Table S1). One of the most common problems for their administration is the difficulty of swallowing them daily by certain population groups (children and the elderly). In such cases, the preparation of modern, easily edible formulations is usually recommended [16]. Of course, the effective release of vitamins from the proposed pharmaceutical form in the oral, gastric, and especially the intestinal cavity is considered important [17]. An additional question that raises an answer is related to the patient’s dietary habits, i.e., whether vitamins should be administered on an empty or full stomach, and what type of food affects their stability. In general, the release of active compounds from a pharmaceutical form in the presence of specific foods such as milk [18] or orange juice [19,20] has been of concern to the scientific community from time to time. E. Fabian and co-workers, in a study, correlated the intake of dairy products with the concentration of vitamins in plasma [21]. Accordingly, E W Nelson and co-workers found that the binding of vitamin B_6_ in orange juice affects its bioavailability [22]. In any case, in order to ensure the accuracy and correctness of these results, the appropriate analytical method adapted to the specific requirements should be applied.

There are a significant number of literature reports (Table 1) for the simultaneous determination of B_1_, B_2_, and B_6_ vitamins in various substrates. The method of choice is usually a reverse-phase HPLC chromatography with gradient elution and UV detection [23,24,25]. The fluorescence detector is proposed for vitamins B_2_ and B_6,_ while B_1_, due to its lack of fluorescence, can only be detected after its oxidation [26]. Another reliable and sensitive detector that has been used in various complex substrates for their detection is the mass detector, which is not recommended for routine analyses due to its complexity and high operating cost.

Considering the circulation of many commercial preparations containing the three vitamins B_1_, B_2_, and B_6_, the present study had a dual role: to propose a flexible and economical HPLC-UV method suitable for their determination in gummies (routine tests for long-term analyses), as well as to develop and apply a sensitive and selective HPLC-FLD method, appropriate for vitamins trace analysis in gastrointestinal fluids (G.I.). More specifically, by applying a three-phase (oral, gastric, and intestinal phase) in vitro digestion protocol [35], the fluorometric method served as a tool for studying their behavior in the presence of orange juice, milk or water. Emphasis was placed on the purification of samples from gastric and intestinal fluids using the Solid Phase Extraction (SPE) technique. The results of the present study can provide useful information both on the integrity of the suggested formulation and on the dietary habits during its administration.

## 2. Results and Discussion

### 2.1. Chromatographic Method Development

The aim of the present chromatographic research was to propose a reliable analytical method for the simultaneous determination of the three analytes (B_1_, B_2,_ and B_6_), isocratically, in a short analysis time. The problems that had to be overcome were the improvement of the B_1_ peak shape and the selection of the appropriate stationary and mobile phase to achieve the elution of the first peak (B_6_) after the solvent front and the last (B_2_ or B_1_) as soon as possible. In order to select the optimal stationary phase, different types of reversed-phase columns (-phenyl, -C18, -CN and Aqua) were tested under various analytical conditions. In Table S2, a summary of all experimental investigations is presented. Phenyl-ACE^®^ (150 mm × 4.6 mm, 5 µm), C18 Supelco Discovery^®^ HS, 250 mm × 4.6 mm, 5 µm (Darmstadt, Germany), and CN Waters Spherisorb^®^, 250 mm × 4.6 mm, 5 µm (Milford, MA, USA) columns were rejected. Optimal performance was achieved with the Aqua Evosphere Fortis^®^, 250 cm × 4.6 mm, 5 µm (St. John’s, NL, Canada) column, which gave the best chromatographic peaks and separations in a short run time.

Methanol, at a ratio of 30%, was used as the organic solvent in the mobile phase instead of acetonitrile, mainly for ecological and economic reasons. Also, due to the high eluting power of acetonitrile, it should not participate at all in the mobile phase, so that B_6_ would elute after the solvent front. Such a mobile phase, however, although relatively compatible with the column, elutes B_1_, B_2_ in very long times. Another curious observation was the fact that small changes in the mobile phase in the organic solvent affected B_2_ to a much greater extent compared to the other two analytes.

To determine the optimal pH value of the mobile phase, various tests were performed, using phosphate buffer solutions and taking into account the properties (Table S3) and chemical structure (Figure S2) of the analytes. Although based on the pKa values of the analytes, their retention time should be affected by changes in pH (2.5–7), this was not observed to a significant extent in the Aqua column, except in the case of vitamin B_1_. Therefore, the research focused on B_1_, which in an acidic environment (H_2_O/FA 0.2%, pH ≈ 2.5), even with a mobile phase containing only 2% methanol, elutes with the solvent front. By increasing the pH, its retention on the column increases proportionally, resulting in it appearing in different positions with respect to the other two peaks. Among the pH values examined (4.5, 4.95, 5.15, 5.8, and 7), 4.95 was chosen as optimal, because B_1_ elutes between the other two vitamins (Rs > 3.3).

The choice of the appropriate diluent for the final samples (before their injection into the HPLC) was based on the solubility of the analytes (Table S3) [36,37], their stability in the various solvents, and the shape of their chromatographic peaks. Thus, the use of 100% methanol was rejected due to limited solubility and poor quality of the chromatograms (peaks with tails). Regarding the stability of the substances, according to the study that follows (Section 2.2), there was no significant limitation. Therefore, the mixtures: H_2_O-MeOH 50:50 *v*/*v* for HPLC-UV and H_2_O-MeOH with FA 0.1%, 50:50 *v*/*v* for HPLC-FLD method were, respectively, chosen as the appropriate diluent, to be compatible with the extraction solvent of the samples (gummies and GI fluids).

After the investigation was complete, a mixture of two phases (A): 20 mM NaH_2_PO_4_ buffer solution at pH 4.95, and (B) methanol, 70:30 *v*/*v* in isocratic elution, was used as the optimal mobile phase. The flow rate was 0.9 mL/min, the injection volume 30 μL, and the column temperature 40 °C, so as not to create pressure problems. For the quantification of B_1_, B_2_, and B_6_ in the pharmaceutical formulation (gummy), a UV detector was used. Based on the literature and the UV spectra obtained from the DAD detector (Figure S1a), B_1_ exhibits maximum absorption at 232 nm, B_2_ at 267 nm, and B_6_ at 220 nm. Similarly, in the case of the fluorometric detector, the excitation and emission wavelengths were, respectively, 385/450 nm for B_1_, 460/525 nm for B_2_, and 290/390 nm for B_6_ (Figure S1b).

### 2.2. Stability Study of B_1_, B_2_, and B_6_ in Different Solvents

The stability study was deemed necessary in order to select the appropriate diluents for both the initial solutions and those resulting from the final dilutions of the samples. At the same time, it had to be examined whether the proposed diluents would maintain the signal intensity (AUC) of the analytes stable for at least 6 h (T = 25 °C), which is usually required to complete an analysis cycle. Thus, mixed standard solutions of B_1_, B_2_, and B_6_ (40 μg/mL) were prepared in four different solvents H_2_O, methanol (MeOH), MeOH-H_2_O 50:50 *v*/*v* and 20 mM NaH_2_PO_4_ buffer solution pH 4.95. The samples were stored in a natural environment and analyzed at regular intervals.

**Figure 1:**
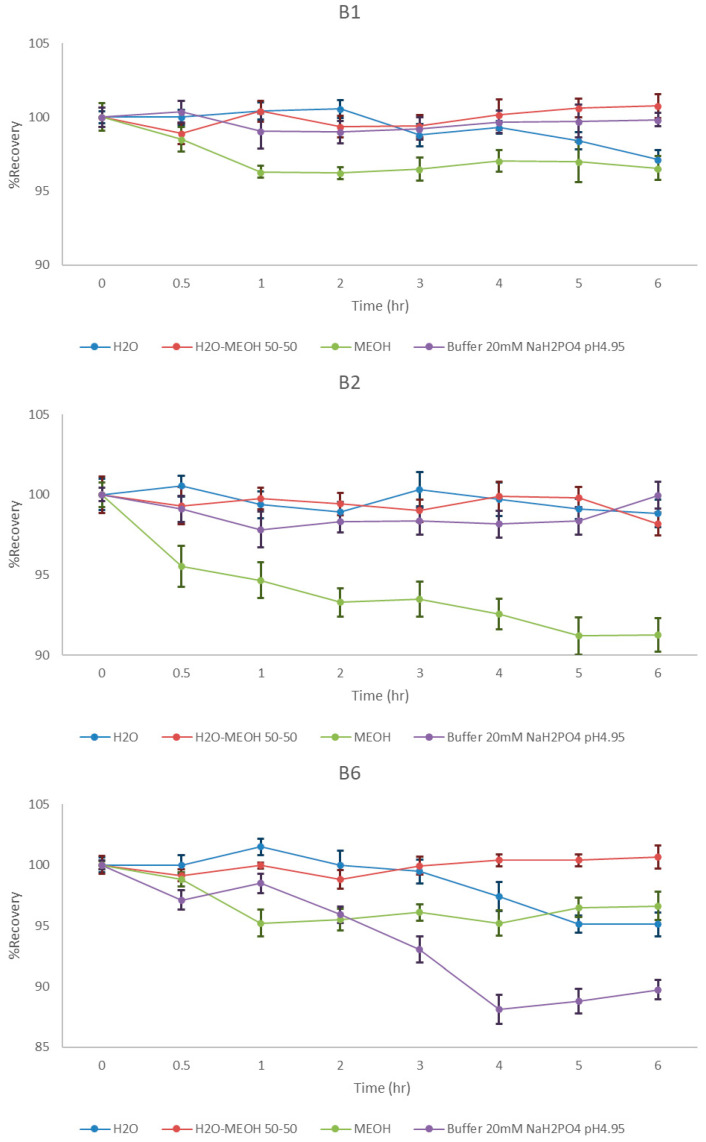
Stability study diagrams of B_1_, B_2_, B_6_ in different solvents, *n* = 3, RSD < 1.5 (blue: H_2_O, red: H_2_O-MeOH 1:1, green: MeOH and purple: buffer NaH_2_PO_4_ 20 mM, pH 4.95).

Based on the results depicted in the stability plots (Figure 1), a significant decrease in AUC values (<90%) of B_6_ was observed after 4 h when phosphate buffer was used, as well as a relative decrease in signal for all analytes in methanol. The other two solvents gave stable signals and could be used as diluents. Finally, considering the good appearance of the chromatograms (narrower and more isometric peaks), the methanol/water ratio of 1:1 was chosen as the optimal solvent in the final dilutions of the samples.

### 2.3. Derivatization Procedure

**Figure 2:**
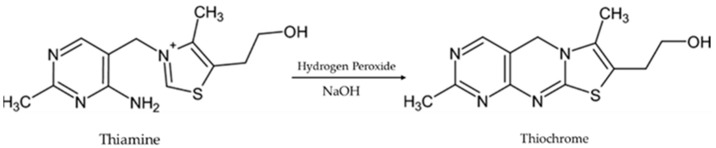
Oxidation reaction of thiamine to thiochrome.

As vitamins B_6_ and B_2_ are natively fluorescent, they do not require further processing for their fluorometric detection and quantification. However, for B_1_, although it does not fluoresce, it gives a strong signal in its oxidized form (thiochrome) [38,39,40]. In one of the most prevalent versions, the derivatization/oxidation of B_1_ can be achieved (within 5 min) with hydrogen peroxide in the presence of sodium hydroxide at ambient temperature (Figure 2).

However, since derivatization/oxidation reactions are usually unstable, further investigation of the most important factors (solvents used, temperature, pH, and reaction time) that may influence the result was carried out. At the same time, it was examined whether the two other active substances, B_6_ and B_1,_ maintained their signal stability under the respective oxidation conditions.

#### 2.3.1. Temperature

Temperature can significantly affect the kinetics of a reaction. Typically, increasing the temperature accelerates the reaction rate, contributing positively to the production of the desired product, while decreasing it slows down or even stops [41,42].

In the present experimental conditions, four different temperatures (−18 °C, 4 °C, 25 °C, and 70 °C) were investigated in order to determine the optimum one that would ensure a high and stable value in signal intensity. To perform the experiments, 0.8 mL standard solution with B_1_, B_2,_ and B_6_ (160 ng/mL) was prepared, which also contained 0.1 mL H_2_O_2_ 30% and 0.1 mL NaOH 5M. The sample remained for 5 min at 25 °C in order to ensure the oxidation reaction of thiamine to thiochrome (Figure 2). Then, part of the standard solution was divided into four HPLC vials and each of them was maintained (for 15 min) at four different temperatures: −18 °C, 4 °C, 25 °C (room temperature), and 70 °C. After the lapse, the samples were analyzed with HPLC-FLD to measure their signal. In the case of B_6_, no alterations were observed at the four temperatures. Vitamin B_2_ remained almost constant at the 3 low temperatures, but at 70 °C it showed a significant drop (sub-doubled). Accordingly, in vitamin B_1_, the greatest destruction was observed at the highest temperature, while at the lower ones its degradation rate decreased (Table S4). For the two low temperatures (−15 to 4 °C), the resistance of the samples over time was tested, and it was found that between 1.5 and 6 h the vitamins gave a fairly stable signal (AUC). In this timeframe, the HPLC-FLD method can be used for long-term routine analyses.

**Figure 3:**
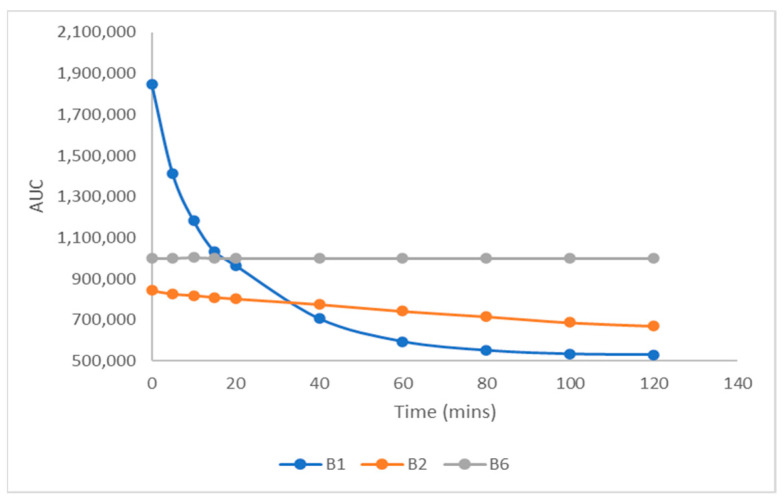
Degradation kinetics of thiochrome, B2, and B_6_ at 25 °C.

Finally, to determine whether or not it is possible to keep the oxidized sample in a simple rack/HPLC for long-term analysis, the same vial (with standard oxidized solution of B_1_, B_2,_ and B_6_) remained at room temperature (25 °C) and was analyzed at regular time intervals. According to the results, only the B_6_ vitamin gave a stable signal in all cases, whereas B_2_ gradually showed a loss of about 15%, and B_1_ was destroyed at a rate of about 80% (Figure 3).

Since maintaining a stable derivative for as long as possible is desirable, two attempts were made to terminate the reaction, one by adding an antioxidant (ascorbic acid) [43] and one by lowering the pH (with HCl 2M). The addition of 0.1 mL of ascorbic acid 0.015–1.5 M, as an antioxidant, led to a strong decrease in the signal from the highest to the lowest concentrations. Accordingly, the gradual decrease in pH, by adding different volumes of 50–150 μL HCl 2M, did not give satisfactory results. Amounts greater than 100 μL of HCl 2M caused the disappearance of the signal, while at lower volumes, the signal was inversely proportional but unstable and of low intensity.

Since none of the proposed methods ensured reproducibility of the results, it was decided to either analyze the samples exactly 5 min after the oxidation reaction or to store them in the refrigerator and analyze them between 1.5 and 6 h.

#### 2.3.2. Diluents

The oxidation reaction of B_1_ in the presence of B_2_ and B_6_ was performed, after the treatment of the sample, in a vial, 5 min before its injection into the HPLC. More specifically, 0.2 mL of 15% aqueous H_2_O_2_ solution at alkaline pH (1:1 30% H_2_0_2_: NaOH 5M) was added to 0.8 mL of a standard solution of the three analytes. Given that 80% of the final sample consists of the diluent of the three analytes, it was considered necessary to study its effect on the signal intensity. Between water and methanol, the second solvent gave a slightly larger peak area for B_1_ (AUC_methanol_ = 1.17 × AUC_water_) and the same signal for the other two compounds, while the signal of the mixtures methanol-water 1:1 *v*/*v*, MeOH with FA 0.1% and MeOH with FA 0.1%–H_2_O was like that of pure methanol. Considering the high solubility of the analytes in water, the good quality of the chromatogram and the fact that the elution solvent in SPE is MeOH with FA 0.1%, the mixture of MeOH with FA 0.1%-H_2_O 1:1 *v*/*v* was chosen as the optimal diluent.

#### 2.3.3. Effect of pH on the Oxidation Reaction

According to literature reports, to carry out the oxidation of thiamine to thiochrome, pH values greater than 8.0 are required, while its maximum fluorescence intensity is at pH values between 12 and 13 [40]. In the present experimental conditions, it was found that the pH of the sample must be greater than 11, while the maximum reaction yield was achieved at pH = 13. The pH adjustment was investigated by adding two different solutions: NaOH 5M or borate buffer. In the case of the NaOH 5M solution, the peak area of B_1_ was twice larger than that with the borate buffer, while the kinetics of the product decomposition were similar in both cases.

### 2.4. Method Validation

Two HPLC methods were proposed, one for the determination of vitamins B_1_, B_2_, and B_6_ in gummies using a UV/DAD detector and the second one in gastric and intestinal fluids using a FLD detector. Both were validated to meet the requirements described in ICH Q2 (R2) [44] according to the following procedures (Section 2.4.1, Section 2.4.2, Section 2.4.3, Section 2.4.4, Section 2.4.5, Section 2.4.6).

#### 2.4.1. System Suitability

Since a basic prerequisite for starting a routine analysis is the suitability of the system, the corresponding test was performed (Table 2).

#### 2.4.2. Selectivity

**Figure 4:**
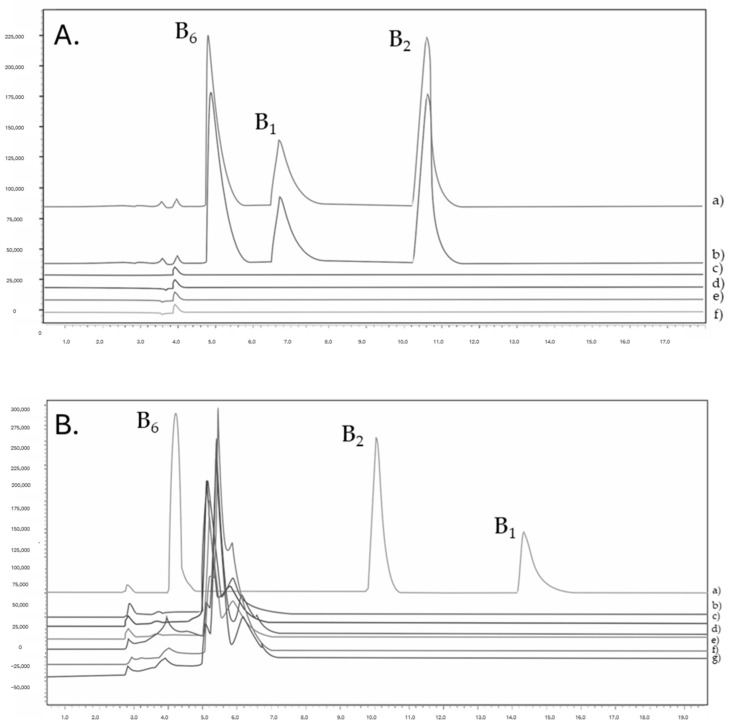
(**A**). Chromatograms with DAD of (a) standard solution, (b) sample extracted from pharmaceutical gummies (with APIs), (c) blank of gastric fluid, (d) blank of intestinal fluid, (e) blank of diluent, and (f) blank of pharmaceutical gummies recovery (no APIs). (**B**) Chromatograms with FLD of (a) standard solution after derivatization, (b) blank of gastric fluid with milk, (c) blank of gastric fluid with water, (d) blank of gastric fluid with orange juice, (e) blank of intestine fluid with milk, (f) blank of intestinal fluid with water, and (g) blank of intestinal fluid with orange juice.

The selectivity of the method was verified by analyzing blank samples (extracted from gummies, gastric and intestinal fluids, gastric fluids with orange juice/water/milk, and intestinal fluid with orange juice/water/milk), unspiked and spiked with vitamins B_1_, B_2,_ and B_6_. Both methods demonstrated effective chromatographic separation of the three active ingredients (APIs), as no contamination interferences (extra peaks) were observed in the retention times of the analytes (Figure 4).

Also notable is the difference between the elution order of the three analytes in the two methods. As derivatization leads to a fluorescent, more lipophilic product (thiochrome), its retention time increases, leading to a different elution order of the analytes.

Additionally, to ensure that both the autosampler and the column are free of contamination, after three consecutive injections of a high concentration sample, a blank sample was analyzed. The chromatogram showed no additional peaks.

#### 2.4.3. Linearity and LOD-LOQ

The linearity of the methods was examined at six concentration levels (3 replicates) for each vitamin. From the results obtained (AUC values), calibration curves were calculated, the characteristics of which are reported in Table 3. The good linearity of the method for each analyte was additionally assessed based on the % y-intercept values (intercept value × 100/100% response), which should be <2%. The limit of detection (LOD) and quantitation (LOQ) were estimated via the following equations [45]:

LOQ = 10 × Sy/x/Slope

LOD = 3 × Sy/x/Slope

where Sy/x is the residual standard deviation and slope is the (x) variable of the calibration curve.

#### 2.4.4. Precision-Repeatability

The intra-day (repeatability) and inter-day precision of the validated methods were examined. Table 4 summarizes the results, expressed as %RSD values. Intra-day and inter-day precision were evaluated by performing triplicate analyses within one day and over three consecutive days, at three levels of sample concentration (low, medium, high).

#### 2.4.5. Accuracy

To verify the accuracy, six samples with B_1_, B_2_, and B_6_ of known concentrations were prepared and analyzed. Each concentration was then calculated from the calibration curve equation and compared to the actual (%Recovery). Both methods were reliable since the mean % recovery values were <100 ± 3% for UV and FLD detectors. (Table S5).

#### 2.4.6. Robustness

To assess the robustness of the two chromatographic systems, the consequences of small modifications to their operating conditions were examined and evaluated in terms of the tailing factor (Tf) and the peak area (AUC) (Table 5).

According to the % RSD values, it was found that the methods were robust to small modifications of temperature and λ_max_ for both detectors. However, they do not show robustness to small changes in the flow rate of the mobile phase, which was expected, especially for the FLD detector. As a possible practice to address a flow rate instability problem, the use of a flow stabilizer could be considered.

### 2.5. Formulation Studies

The usual recommended daily intake of the three vitamins (B_1_, B_2,_ and B_6_) is approximately 1 mg for adults and 0.5 mg for children [46,47]. In the present experimental conditions, a dosage formulation of 0.5 mg was prepared in the form of gummies. The main component of the formulation was the aqueous carrier, which, being in sufficient quantity, ensured the complete dissolution of the active ingredients [48,49]. Crystalline sugar or alternatively liquid stevia (4 drops versus 5 g) was used as a sweetener. Gelatin powder was used to produce jelly, which is easier to handle compared to the corresponding gelatin sheets [50,51,52]. Finally, the addition of chemical dyes was not necessary, as vitamin B_2_ is colored and gives a natural yellow hue. In total, each dosage unit contained 0.5 mg B_1_, 0.5 mg B_2_, 0.5 mg B_6_, 533 mg gelatin, 1 g sucrose, and 5.2 g water.

**Figure 5:**
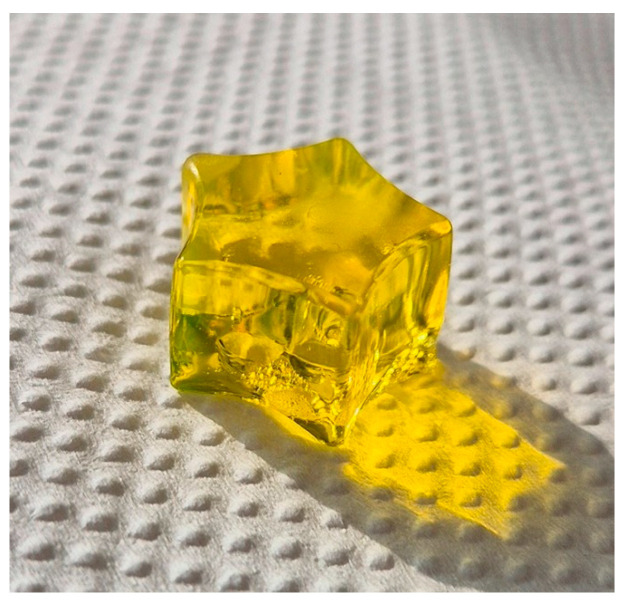
Gelatin preparation with 0.5mg of B_1_, B_2,_ and B_6,_ respectively.

To complete all planned experimental studies, 15 units were prepared according to the following procedure: Initially, 8 g of gelatin powder were accurately weighed and added to 8 g of water (3–4 min) in order to swell the gelatin. Then, 7.5 mg of each vitamin was dissolved in a separate container in 70 mL of water with the aid of stirring and ultrasound. This was followed by the addition of granulated sugar (15 g) with parallel stirring and heating (water bath at 40 °C). Finally, the gelatin (which had been swollen by hydration in a hot aqueous solution) was added, and the mixture was stirred until completely dissolved (water bath at 40 °C). The final mixture (101.02 g) was accurately divided into 15 pre-weighed silicone molds, which were placed in a refrigerator at 2 °C (Figure 5).

#### 2.5.1. Sample Pretreatment

For the quantitative determination of B_1_, B_2,_ and B_6_ in the gummy formulation, the appropriate solvent was chosen, which would selectively extract only the active ingredients (liquid-solid extraction) and not the substrate components. For this reason, although water is the optimal solvent for APIs, it was rejected, and methanol (50 mL/formulation) was used instead because it does not dissolve sucrose, and when it comes into contact with gelatin, it solidifies [50]. At the same time, the stability of the active ingredients under the formulation processing conditions, i.e., methanol as solvent, heating to 35 °C, and sonication (Table S6), was examined. Given that after 30 min of sample processing, vitamin B2 is destroyed, it was decided to simultaneously subject the standard solution to the same procedure.

Taking into account all relevant information, the following sample pretreatment was applied: A single dosing unit (6.7 g) was placed in a glass beaker and heated in a water bath (40 °C) until it melted. While the sample was sonicated (35 °C), 50 mL of methanol was added dropwise, so as to avoid solidification of the gelatin and entrapment of APIs within it. The sample was then sonicated for an additional 15 min (without heating), stirred (5 min), and placed in the freezer (1 h). Part of the supernatant was centrifuged (5000 rpm for 15 min), and 2 mL were quantitatively transferred to a 10 mL volumetric flask, which was filled to the mark with H_2_O-MeOH 50:50 *v*/*v*. The final solution was analyzed by HPLC-UV. The proposed method was applied to 5 units (gummies), and the % recovery of the analytes was calculated. According to the results, the % recoveries were found to be 100.02% (%RSD = 2.66) for B_6_, 99.89% for B_1_ (%RSD = 4.22) and 99.9% (%RSD = 2.18) for B_2_ (Table S7).

#### 2.5.2. Formulation Stability Study

To evaluate the compatibility of the APIs with the substrate, a short-term stability study of the formulation was performed. More specifically, the quantitative determination of the three vitamins in the formulation was performed on the 1st, 2nd, 7th, and 28th day, while it was stored at 2 °C. By the 7th day, their recovery was >98.8%, while on the 28th day, for B_1_ and B_6_ it was >97.5% and for B_2_ it was equal to 96.5%.

### 2.6. In Vitro Digestion Protocol

To study the behavior and release rate of the three vitamins incorporated in the gummies, three simulated digestive fluids were used: Salivary (SSF), Gastric (SGF), and Intestinal (SIF). The same in vitro digestion protocol was applied to three separate replicates differing only in the oral stage, to which either water, orange juice, or milk was additionally added.

Particular importance was given to the selection of the appropriate analytical method, which must be selective and sensitive. As such, an HPLC with a fluorometric detector was used, while the sample purification was performed by a solid phase extraction [53].

#### 2.6.1. Samples Pretreatment

International literature reports various methods for the extraction of B complex from biological fluids, food, or seawater. These were usually achieved by solid phase extraction (SPE) technique, in C18 cartridges, using methanol [54], methanol/water [28], ethanol/water [29], and methanol-phosphate buffer pH 3.0, as eluent [55].

Under the present conditions, for the optimization of the suggested solid phase procedure, various SPE cartridges and elution solvents were tested and evaluated. Using Supelco SupercleanTM ENVI-18 (Bellefonte, PA, USA) and WATERS OASIS cartridges (Milford, MA, USA) (500 mg/3 mL), the retention of the three analytes was strong, and more than 3 mL of methanol was needed for their elution. In contrast, in the single-layer cartridge, Empore C18-SD (Oxford, PA, USA) (7 mm/3 mL), no vitamins could be retained at all, while in the corresponding two-layer cartridge, their retention was about 80%. The optimal SPE conditions include a single-layer and two-layer Empore C18-SD (7 mm/3 mL) cartridges, connected together in series, which have been conditioned with 1 mL of methanol and 2 mL of water. This technique significantly increased the retention of the cartridges both individually and cumulatively. This is probably explained by the fact that when the sample is loaded, the cartridges are saturated mainly by the analytes and to a lesser extent (about 20%) by ingredients of the SGF and SIF fluids. Essentially, the first cartridge acts as a cleaning filter, binding lipids and proteins of the carrier, allowing the next one to use all the retention sites with the analytes, leading to higher %Recoveries. The samples derived from the gastric fluids, before being loaded into the cartridges, were alkalized with 0.01M NaOH (1:1 dilution) to prevent ionization and elution of B_1_ [29]. After loading the sample (1 mL sample with B_1_, B_2_, and B_6_ of 15 μg /mL), the vitamins were eluted with 1 mL of MeOH:FA 0.1%. The eluate was collected, diluted with H_2_O (1:1 *v*/*v*), and analyzed. %Recoveries values in the SGF samples were 103.1 (RSD 3.1%) for B_1_, 99.6 (RSD 2.2%) for B_2,_ and 100.3 (RSD 2.4%) for B_6_. Respectively, for the SIF samples, the % Recoveries were: 99.4 (RSD 5.2%) for B_1_, 109.7 (RSD 3.5%) for B_2,_ and 95.2 (RSD 3.8%) for B_6_. The presence of water, orange juice, or milk did not have a significant influence on the %Recoveries values of the analytes (Table S8).

#### 2.6.2. Stability Study of B_1_, B_2_, and B_6_ in Digestive Fluids

The three vitamins are sensitive to various environmental conditions, such as the effect of temperature and light, or to very high or low pH values. More specifically, B_1_ is unstable in strongly acidic and alkaline solvents, especially when subjected to parallel heating [56]. Correspondingly, B_2_ is photosensitive and even though it presents relative stability at pH 5–6, at alkaline pH it is mainly denatured into lumichrome and lumiflavin [57]. Finally, pH (especially at high values) in combination with temperature and light play an important role in the stability of pyridoxine [58].

**Figure 6:**
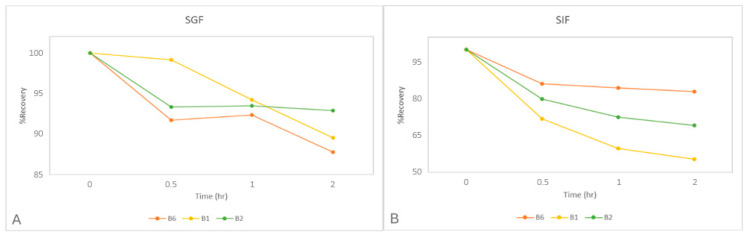
Stability study of B_1_, B_2,_ and B_6_ in (**A**) SGF and (**B**) SIF fluids.

Although several literature reports provide data on their stability, it was considered necessary to conduct an additional study in digestive fluids in order to better characterize their behavior. For the study, two separate, standard solutions of vitamins B_1_, B_2_, and B_6_ (15 μg/mL) were prepared, one in SGF (pH:3) and the other in SIF (pH:7). After being placed in a water bath at 37 °C, sampling, purification, and analysis of the samples by HPLC were performed at regular intervals (Figure 6).

According to the results, at the end of the gastric stage (after 2 h), the three vitamins remain relatively stable (%Recovery > 88%) with a clear predominance of B_2_ (93%). On the contrary, in the intestinal stage, a high rate of alteration was observed, especially in B_1_ (55% remains stable), while B_6_ remains stable by 83%. In summary, the vitamin that is mostly destroyed is B_1_ (total losses 55%), B_6_ is destroyed less (total losses 27%), while B_2_ shows intermediate stability (total losses 37%).

#### 2.6.3. Digestion Protocol Results and Discussion

**Figure 7:**
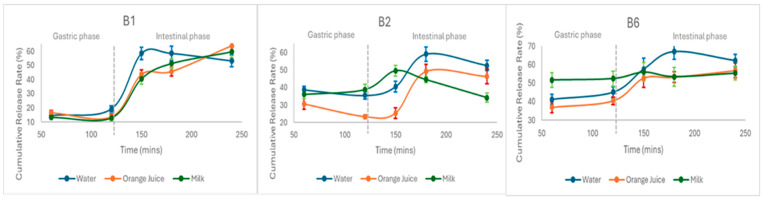
Cumulative percentage of the initial drug content at the gastric and intestinal phase.

The release rate of the three vitamins from the jelly was recorded and studied by applying the full digestive protocol (oral cavity, stomach, and small intestine) to three different dietary habits (water, orange juice, or milk). The amount number of APIs released in each phase was measured and reported as the cumulative percentage of the initial drug content (Figure 7).

In general, their release depends on their solubility in gastrointestinal fluids and on the food web they are contained in. Factors such as acidity (orange juice), fat content, protein, casein, and calcium (milk), or the presence of polysaccharides/fiber (orange juice) can modify their solubility, digestive behavior, and bioavailability [59,60].

In the present experimental conditions (Figure 7) it appears that the pattern of B_1_, B_2_, and B_6_ behavior was generally similar in water, orange juice, and milk. More specifically, it was found that when the experiments were carried out in the gastric phase with the addition of orange juice, the three vitamins were released in small amounts, ranging up to 16% for B_1,_ while for the other two, it was less than 40%. B_1_ presents the same amounts in the stomach, regardless of the presence of water, milk, or orange juice, while B_2_ and B_6_ had the lowest release in orange juice and the highest in milk.

Given that the degree of degradation of B_1_, B_2_, and B_6_ in gastric fluid is low (Figure 6A) and that their solubility in water is high (Table S3), it is possible that their low release rates are due to their entrapment by the gummies substrate. Indeed, gelatin is a protein derived from collagen and consists of amino acid chains, which at acidic pH values could couple with the vitamins (hydrogen bonds) and precipitate. Of course, during their transport to the small intestine, their release rate increases (B_1_ > 53%, B_2_ > 34% and B_6_ > 55%), although a part of them is destroyed at alkaline pH. This increase is probably due to their release from gelatin, which is probably favored at higher pH values.

At the end of the protocol, the release rates for each vitamin appear to be almost similar and independent of the dietary habits (water, orange juice, and milk). It could, of course, be said that B_1_ is favored by the parallel administration of orange juice, while for the other two vitamins, their release was greater in water. This is also demonstrated by the ratio of the amount of active ingredients found in the last sampling compared to that found during the sediment analysis (Table 6).

## 3. Materials and Methods

### 3.1. Instruments and Equipment

Chromatographic separation was executed with a Shimadzu (Tokyo, Japan) HPLC arrangement consisting of two LC-20AD pumps, a DGU 14A degasser, a SIL-10AD autosampler (injection volume, 30 μL), and a CTO-20A column oven (temperature 40 °C). Two detectors were used: an ultraviolet photodiode array (UV-DAD (SPD-M20A) and a fluorescence (FLD), RF20-A (Shimadzu, Tokyo, Japan). The FLD detector was set at Gain: ×4 and at high sensitivity. The analytical column was a reversed-phase Aqua Evosphere Fortis^®^, 250 cm × 4.6 mm, 5 μm (St. John’s, NL, Canada). The isocratic elution was performed with two mobile phases A: phosphate buffer (pH = 4.95), and B: methanol at a ratio of 70:30. The Flow rate was set at 0.9 mL/min. The LC solution software (version 1.25 SP4) was used for data processing.

For the in vitro digestion protocol, a Thermostatic Shaking Water Bath set at 37 °C from Witeg (Wertheim, Germany) was utilized. Furthermore, the samples were centrifuged in a Labofuge^®^ 400 R centrifuge (Waltham, MA, USA).

For the FLD spectra of the analytes, a RF-5301PC Spectrofluorophotometer, Shimadzu (Tokyo, Japan) was used.

### 3.2. Reagents and Solvents

Methanol (MeOH) was of HPLC grade and obtained from Honeywell (Frankfurt, Germany), whereas formic acid (FA) was from Sigma Aldrich (St. Louis, MO, USA). Water was of high purity (18.2 MΩ cm resistivity) and produced by a B30 water purification system (Adrona SIA, Riga, Latvia). Hydrogen peroxide, 30% reagent (Scharlau, Barcelona, Spain), and sodium hydroxide, NaOH (A.C.E.F., Piacenza, Italy) were used for the derivatization procedure.

The analytes riboflavin (B_2_), pyridoxine hydrochloride (B_6_), and thiamine hydrochloride (B_1_) with purity >98.0% were purchased from TCI (Zwijndrecht, Belgium) (Figure S2). Gelatine, sugar, milk, and orange juice were obtained from a local shop in Thessaloniki.

### 3.3. Solutions

#### 3.3.1. Stock Solutions

A total of 5.00 mg of vitamin B_2_ was accurately weighed and dissolved in 100 mL of H_2_O. Similarly, 5.00 mg of B_1_ and B_2_ were dissolved in two separate 10 mL volumetric flasks, which were filled with MeOH. Subsequently, a mixed solution of the three analytes was prepared, from which, with appropriate dilutions, two series of six standard solutions were obtained (Table 3). The samples were used for the calibration of the two methods, HPLC-DAD (diluent MeOH-H_2_O 1:1 *v*/*v*) and HPLC-FLD (diluent MeOH with Formic acid 0.1%-H_2_O 1:1 *v*/*v*), respectively.

#### 3.3.2. Derivatization Solutions

For the derivatization/oxidation process, 0.8 mL of standard solution with B_1_, B_2_, and B_6_ was mixed with 0.1 mL of 30% H_2_O_2_ and 0.1 mL of NaOH 5M. To prepare NaOH 5M, 2.5 g of NaOH beads were dissolved in 10 mL of H_2_O.

#### 3.3.3. Stimulated Fluids

To perform the in vitro digestion protocol, three digestion fluids were prepared. The preparation procedure of Simulated Salivary (SSF), Gastric (SGF), and Intestinal (SIF) Fluids is described in detail in Text S1 [14].

### 3.4. Gummies Preparation

Two separate containers were used to form 15 gummy dosage units. In the first step, 8 g of gelatin (powder) was moistened with 8 g of water. At the same time, 7.5 mg of each B-complex vitamin and 15 g of sugar were accurately weighed and transferred to a beaker (placed in a water bath at 40 °C) where they were dissolved by stirring and ultrasound. The two mixtures were mixed (under stirring) in the thermostated water bath, and the final product was distributed into 15 pre-weighed (before and after) silicone molds. The 15 dosage units remained at 4 °C to solidify and maintain stability.

### 3.5. Pretreatment of the Formulation Before Analysis

Once a dosage form (gummy) was heated (water bath 40 °C) until it melted, 50 mL of methanol were added dropwise, in a temperature-controlled ultrasonic bath (35 °C). Then, the sample remained without heating in the ultrasonic bath for another 15 min, was stirred (5 min), placed at −18 °C (1 h) and part of the supernatant was centrifuged (5000 rpm for 15 min). Finally, after being diluted 1:5 (diluent: H_2_O-MeOH 50:50 *v*/*v*) it was analyzed with the proposed HPLC-DAD method.

### 3.6. In Vitro Digestion Protocol

The same digestion protocol was performed three times separately to study the effect of the H_2_O, orange juice, and milk (three replicates in each case), according to the following procedure. A dosage unit (gummy) was placed in a plastic centrifuge tube (50 mL) to which 5 mL of water, orange juice, or milk and 5 mL of SSF were added and mixed in the stirred water bath (2 min at 37 °C). After the oral phase was completed, 10 mL of SGF was added and stirring was continued. The gastric phase lasted 2 h, using the same stirred water bath, thermostatically controlled at 37 °C. In the final stage (intestinal phase), the gastric fluid was mixed with 19.85 mL of intestinal fluid (pH 7) under the same experimental conditions for 2 h. During the in vitro digestion protocol, samples (500 μL) were taken at 1 and 2 h (in the gastric phase) and at 0.5, 1, and 2 h (in the intestinal phase). Subsequently, three blank samples were prepared, one for each liquid (water, orange juice, and milk) containing the formulation vehicle without the APIs (unspiked gummies). These were subjected to the entire digestion protocol as described for the samples and analyzed with the proposed method.

#### 3.6.1. SPE Procedure

To 0.5 mL of the sample, derived from the digestion protocol, was added either 0.5 mL of H_2_O (intestinal phase) or 0.5 mL of 0.01M NaOH solution (gastric phase). Subsequently, the sample was loaded into a device with two cartridges connected in series (a single-layer and a two-layer Empore C18-SD (7 mm/3 mL), which have been conditioned with 1 mL of methanol and 2 mL of water.

For the elution of the APIs, 1 mL of MeOH with FA 0.1% was used. The eluate, after being diluted with 1 mL of H_2_O, was filtered (0.45 μm PTFE filter) and analyzed based on the proposed HPLC-FLD method.

#### 3.6.2. Sediment Reconstitution

Once the in vitro digestion protocol was completed, the determination of the three vitamins in the precipitate was performed. The precipitate and supernatant were collected, frozen (20 min), and centrifuged (at 5000 °C, 10 min, two cycles). The supernatant liquid was decanted to obtain only the precipitate, which was then subjected to the same processing procedure as that of the preparation.

## 4. Conclusions

The application of two analytical methods for the determination of vitamins B_1_, B_2_, and B_6_ in jelly formulations was successfully proposed. HPLC-DAD, as it is more flexible, could be used in routine tests, and HPLC-FLD in specialized and sensitive determinations. In both cases, the use of an Aqua Evosphere Fortis^®^ column (250 cm × 4.6 mm, 5 μm) is recommended for isocratic elution of the analytes. Sample cleanup by solid–liquid or solid-phase extraction is considered a necessary pretreatment step. The validated methods were successfully applied, providing information on the integrity/stability of the pharmaceutical formulation (gummies) as well as on the release rate of APIs in the GI (digestion protocol). According to the results obtained from an in vitro digestion protocol, it was found that there is no significant difference in the release rates of the vitamins when they are administered simultaneously with different dietary habits (water, orange juice, or milk). Only a slight superiority was observed in the release of vitamins B_2_ and B_6_ with water and B_1_ with orange juice.

## Figures and Tables

**Table 1:** Literature review for the simultaneous determination of vitamins B_1_, B_2,_ and B_6_.

Samples (Reference)	Method	Stationary-Mobile Phase	Sample Preparation/Extraction Method	LOD
Dried blood spots (DBSs) [27]	LC-MS/MS	ACE^®^ C8 Column, 4.6 × 100 mm, 5 μm (Wrotham, UK) Gradient: (A) H_2_O/formic acid 0.1% (*v*/*v*) and (B) acetonitrile	hydration (trichloroacetic acid), sonication, centrifugation	B_1_: 0.5 ng/mL B_2_: 0.2 ng/mL B_6_: 0.5 ng/mL
Foods [28]	LC/ESI-MS/MS	Avantor^®^ Alltima C18, 250 mm × 4.6 mm, 5 mm (Chadds Ford, PA, USA) Gradient: (A) acetonitrile with 5 mmol/L formic acid and (B) water with 5 mmol/L formic acid	SPE-0.5 g, C18, elution with 14 mL of EtOH/H_2_O 1:1	B_1_: 2.0–12.9 ng/g B_2_: 4.0–6.2 ng/g B_6_: 0.9–11.0 ng/g
Seawater [29]	UPLC/ESI-MS	UPLC HSS Cyano Column Waters Acquity^®^, 2.1 × 100 mm, 1.8 μm (Milford, MA, USA) Gradient: (A) 20 mM ammonium formate with 0.1% formic acid in water (B) and acetonitrile	C18 SPE (Waters, 35 mL, 10 g resin), samples: at pH 5.5–6.5 with HCl, adjusted to pH 6.5, elution with 40 mL MeOH	B_1_: 0.059 pM B_2_: 0.124 pM B_6_: 0.149 pM
Infant formula and related nutritionals [30]	LC-MS/ MS-ESI	Waters Acquity^®^ BEH C18 Column, 2.1 × 100 mm, 1.7 mm (Milford, MA, USA) Gradient: (A) 20 mM ammonium formate and (B) methanol	1% glacial acid in methanol, centrifugation, 50 mM ammonium formate, filtration	-
Food products (cacao and milk powder, infant food, orange juice powder) [31]	HPLC UV-DAD/FLD	C18 BDS, Thermo Fisher^®^ 100 × 4.6 mm, 3 µm (Waltham, MA, USA) Gradient: (A): 5.84 mM of hexane-1-sulfonic acid sodium: acetonitrile (95:5) with 0.1% triethylamine at pH 2.5 and (B): similar to (A) in 50:50.	Step 1: centrifugation, sonication, evaporation of MeOH, addition 0.1 mL NaOH 0.17 M, Step 2: 0.1 mL H_3_PO_4_ 5 M, sonication, centrifugation Step 1 + Step 2 filtration	B_1_: 16.5 ng/mL B_2_: 1.9 ng/mL B_6_: 1.3 ng/mL
Royal Jelly [26]	HPLC UV-DAD/FLD	Vydac^®^ C18 reversed phase Column, 250 mm × 4.6 mm, 5 µm (Hesperia, CA, USA) Isocratic: hexanesulfonic acid, ammonium hydroxide, acetonitrile and water (0.09:0.05:9.02:90.84) with pH adjusted to 3.6	1 mL 8% trichloroacetic acid, centrifugation, filtration	B_1_: 66.90 ng/mL B_2_: 6.47 ng/mL B_6_: 7.80 ng/mL
Milk Products [32]	HPLC UV-Vis/FLD	C18 Waters Spherisorb^®^ ODS-2 Column, 250 mm × 4.6 mm, 3 µm (Milford, MA, USA) Gradient: (A) phosphate buffer, pH 2.95 (6.8 g KH_2_PO_4_, 1.1 g of 1-octanesulfonic acid, Na salt, and 5 mL of triethylamine in 1 L of H_2_O) and (B) MeOH	sonication, centrifugation, filtration	B_1_: 0.02 μg/mL B_2_: 0.005 μg/mL B_6_: 0.04 μg/mL
Energy drinks [33]	HPLC PDA/FLD	Nova-Pak C18 Column Waters Spherisorb^®^, 150 mm × 3.9 mm, 5 μm (Milford, MA, USA) fitted with μBondapak C18 cartridge guard column Gradient: (A) methanol and (B) 0.05M NaH_2_PO_4_ containing 0.005 M hexanesulfonic acid, pH 3.0	ultrasonic degassing	B_1_: 25 ng/mL B_2_: 8 ng/mL B_6_: 19 ng/mL
Protein Powders [34]	HPLC-FLD	Thermo^®^ Hypersil, Aquasil C18 Column, 4.6 × 150 mm (Waltham, MA, USA) Post-Column Derivatization System: Onyx PCX, Pinnacle PCX Gradient: (A) 4.77 g of Potassium Phosphate Monobasic in 1 L of DI water (pH to 5.9 with KOH) and (B) acetonitrile Post-Column Conditions: 10 g of Sodium Hydroxide in 500 mL of water and add 2 g of Sodium Sulfite	extraction buffer (0.1 N NaOH: pH 2 with H_3_PO_4_), heat at 100 °C, cool, filtration	B_1_: 0.03–10 μg/mL B_2_: 0.03–10 μg/mL B_6_: 0.125–10 μg/mL
Multi-Vitamins Supplements Tablets [34]			blend the tablets, dissolve with water acidified to pH 2.6 with 0.1 N HCl, magnetic stirring, filtration	

**Table 2:** System suitability parameters using (**a**) HPLC/UV-DAD and (**b**) HPLC-FLD.

**(a)**						
**Analytes**	Tr *	Tf *	**K** ^′^ *	Rs *	N *	HETP * **×10^3^ USP**
B_1_	6.2	2.7	2.647	3.35	2090.0	119.616
B_2_	10.0	1.3	4.889	5.97	8036.6	31.108
B_6_	4.4	2.0	1.605	-	4073.3	61.367
**(b)**						
**Analytes**	Tr *	Tf *	K^′^ *	Rs *	N *	HETP * **×10^3^ USP**
B_1_	14.4	2.6	7.525	7.28	12155.0	20.567
B_2_	10.0	1.2	4.920	15.87	9433.0	26.502
B_6_	4.2	2.0	1.487	-	3721.1	67.184

* Tr: Retention Time, Tf: Tailing Factor, K′: Capacity, Rs: Resolution, N: Number of theoretical plates, HETP: Height equivalent of a theoretical plate.

**Table 3:** Linear regression analysis data.

APIs	Concentration	Equation	%y Intercept	(R^2^)	LOD	LOQ
	μg/mL	HPLC-UV *				
B_1_	1.6–40	y = (63,009 ± 553.4)x − 28,095 ± 10,134.4	1.13	0.999	0.5	1.6
B_2_	0.8–20	y = (201,686 ± 1214.0)x − 10,894 ± 11,116.3	0.27	0.999	0.2	0.6
B_6_	0.8–20	y = (130,165 ± 1097.3)x + 1292 ± 10,047.3	0.05	0.999	0.3	0.8
	ng/mL	HPLC-FLD *				
B_1_	60–1600	y = (19,058 ± 305.1)x + 254,796 ± 229,991	0.73	0.999	7.9	24.1
B_2_	4–160	y = (198,712 ± 2208.6)x − 243,031 ± 166,541	0.77	1	0.9	2.8
B_6_	4–160	y = (67,295 ± 965.8)x + 195,017 ± 72,827	1.61	0.999	1.2	3.6

* Concentration expressed in μg/mL for UV and in ng/mL for FLD detector.

**Table 4:** Results for intra- and inter-day precision.

			HPLC-UV *				
APIs	Repeatability		Intermediate Precision				
	Concentration	RSD%	Concentration	1st Day	2nd Day	3rd Day	RSD%
	1.6 (*n* = 3)	0.22	1.6 (*n* = 3)	0.22	1.67	1.77	1.41
B_1_	8 (*n* = 3)	0.36	8 (*n* = 3)	0.36	0.44	0.12	0.74
	40 (*n* = 3)	0.09	40 (*n* = 3)	0.09	0.45	0.49	0.54
	0.8 (*n* = 3)	0.42	0.8 (*n* = 3)	0.42	1.02	0.7	0.75
B_2_	4 (*n* = 3)	0.14	4 (*n* = 3)	0.14	0.18	0.18	0.52
	20 (*n* = 3)	0.3	20 (*n* = 3)	0.3	0.15	0.07	0.32
	0.8 (*n* = 3)	0.48	0.8 (*n* = 3)	0.48	0.65	0.34	0.7
B_6_	4 (*n* = 3)	0.50	4 (*n* = 3)	0.50	1.09	0.36	0.81
	20 (*n* = 3)	0.07	20 (*n* = 3)	0.07	0.53	0.08	0.34
			HPLC-FLD *				
B_1_	60 (*n* = 3)	1.02	60 (*n* = 3)	1.02	1.76	3.05	3.23
	400 (*n* = 3)	2.01	400 (*n* = 3)	2.01	2.93	0.48	2.81
	1600 (*n* = 3)	1.1	1600 (*n* = 3)	1.1	1.67	1.61	1.39
B_2_	4 (*n* = 3)	0.87	4 (*n* = 3)	0.87	1.41	1.82	1.11
	40 (*n* = 3)	1.3	40 (*n* = 3)	1.3	0.84	1.24	1.77
	160 (*n* = 3)	0.46	160 (*n* = 3)	0.46	1.48	0.98	1.81
	4 (*n* = 3)	1.11	4 (*n* = 3)	1.11	0.57	1.76	1.69
B_6_	40 (*n* = 3)	1.96	40 (*n* = 3)	1.96	1.38	1.12	1.79
	160 (*n* = 3)	0.6	160 (*n* = 3)	0.6	1.71	0.39	1.21

* Concentration expressed in μg/mL for UV and in ng/mL for the FLD detector.

**Table 5:** Robustness test.

Parameters	%RSD (UV/FLD)					
	B_1_		B_2_		B_6_	
	AUC	Tf	AUC	Tf	AUC	Tf
Flow rate (±0.1 mL/min)	7.59/4.91	6.11/9.14	11.76/6.88	6.56/13.81	10.22/10.11	4.7/9.51
Temperature (±2 °C)	2.53/0.33	1.97/0.42	0.26/1.2	0.35/0.79	0.78/1.22	0.86/0.85
Mobile phase (±1%) A:B	2.16/1.91	0.72/0.79	0.31/0.22	0.58/0.79	2.61/2.33	3.88/3.91
λ_max_ (±1 nm)	0.58/0.34	0.55/0.29	0.46/0.51	0.1/0.38	0.46/0.77	0.04/0.81

**Table 6:** Ratio of the amount of analyte found in the last sampling of the intestinal phase to that found in the final sediment.

Vitamins	Water		Orange Juice		Milk	
	%Found					
	Intestinal	Sediment	Intestinal	Sediment	Intestinal	Sediment
B_1_	79	21	91	9	85	15
B_2_	73	17	68	22	63	27
B_6_	80	10	79	11	77	13

## Data Availability

Data are contained within the article and Supplementary Materials.

## Supplementary Materials

The following supporting information can be downloaded at: https://www.mdpi.com/article/10.3390/molecules30193902/s1, Table S1. Commercially available B-complex drug formulations; Table S2. Summary table of chromatographic tests; Table S3. Physicochemical properties of vitamins B_1_, B_2_, and B_6_; Figure S1a. UV spectra of vitamins B_1_, B_2,_ and B_6_; Figure S1b. FLD spectra of thiochrome and vitamins B_2_ and B_6_; Table S4. Stability of B_1_, B_2,_ and B_6_ in 15 min after the derivatization procedure at different temperatures; Table S5. Accuracy test for both UV and FLD detectors; Table S6. %Recoveries of methanolic solution of B_1_, B_2_, B_6_, as a function of time, after ultrasound and heating; Table S7. %Recovery of B_1_, B_2_ and B_6_ from the gummy formulation; Table S8. %Recoveries values in SGF and SIF samples after SPE, in the presence of water, orange juice, and milk.; Figure S2. Chemical structure of B_1_, B_2_, B_6_; Text S1: Preparation of in vitro digestion fluids.
