# Supplementary material for: Development and Validation of HPLC-DAD/FLD Methods for the Determination of Vitamins B1, B2, and B6 in Pharmaceutical Gummies and Gastrointestinal Fluids—In Vitro Digestion Studies in Different Nutritional Habits
Source: Molecules. 2025 Sep 26;30(19):3902. doi: 10.3390/molecules30193902 (PMC12525618; doi:10.3390/molecules30193902)
Supplement: Supplementary file 1 [file molecules-30-03902-s001.zip › molecules-3877306-supplementary.pdf]

Supplementary

Development and Validation of HPLC-DAD/FLD Methods for the Determination of Vitamins B1, B2, and B6 in Pharmaceutical Gummies and Gastrointestinal Fluids—In vitro Digestion Studies in Different Nutritional Habits

Georgios Kamaris †, Nikoletta Pantoudi † and Catherine K. Markopoulou *

Laboratory of Pharmaceutical Analysis, Department of Pharmacy, Aristotle University of Thessaloniki,54124 Thessaloniki, Greece; kamarisg@pharm.auth.gr (G.K.); npantou@pharm.auth.gr (N.P.)
* Correspondence: amarkopo@pharm.auth.gr ; Tel.: +30-2310-997665
† These authors contributed equally to this work.

Table S1. Commercially available B-complex drug formulations.

Formulation	Commercial Formulations
Tablets	Solgar B-Complex "100"
Capsules	Pure Encapsulations B-Complex Plus
Effervescence	Juvamine Magnesium and Vitamines B1, B2, B6
Gummies	NEVISS Vitamin B Complex Gummies
Chewable tablets	Nature's Way Alive! Children's Daily Chewable Multivitamin
Liquid	FutuNatura B complex Liquid
Sublingual tablets	Superior Source, Balance B Complex, MicroLingual Instant Dissolve Tablets
Liposomal	Quicksilver Scientific Liposomal Methyl B-Complex
Injectables	Neurobion Injection
Chewing gum	Blockhead Energy Gum Peppermint

Table S2. Summary table of chromatographic tests.

**Stationary phase Aqua Evosphere Fortis® (250 cm × 4.6 mm, 5 μm) elution: isocratic, flow:0.8-0.9 ml/min, T: 40°C, injection volume: 30μl**			
**Mobile phase**	**Mobile phase solvent ratios (%)**	**Diluent**	**Comments**
H_2O/FA 0.2%	100	H_2O	Peak of B_1 with tailing 3 min after solvent front
H_2O/FA 0.2% -MeOH	98-2	H_2O	Peak of B_1 with tailing at the solvent front
H_2O/FA 0.2% -ACN	99-1	H_2O	Double peak of B_1 at the solvent front
H_2O/FA 0.2% -ACN	99-1	H_2O-ACN 90-10	Peak of B_1 with tailing at the solvent front
20mM NaH_2PO_4 pH 4.5 - MeOH	50-50	H_2O	t_{RB6}=4min - peak with tailing<br>t_{RB2}=4.4min - sharp peak
20mM NaH_2PO_4 pH 4.5 - MeOH	80-20	H_2O	t_{RB1}=6.7min - peak with tailing<br>t_{RB2}>22min
20mM NaH_2PO_4 pH 4.5 - MeOH	70-30	H_2O	t_{RB6}=4.5min - peak with tailing<br>t_{RB1}=5.5min - peak with tailing<br>t_{RB2}=10.5min - sharp peak
20mM NaH_2PO_4 pH 4.95 - MeOH	70-30	H_2O	t_{RB6}=4.5min - peak with tailing<br>t_{RB1}=6.2min - peak with tailing (Rs:3.5)<br>t_{RB2}=10min - sharp peak (Rs:6)
20mM Na_2HPO_4 pH 4.95 - MeOH	70-30	H_2O-MeOH 50-50	Same with diluent H2O - final method
20mM Na_2HPO_4 pH 4.95 - MeOH	70-30	MeOH	Peaks with tailing
20mM Na_2HPO_4 pH 4.95 - MeOH	70-30	H_2O-ACN 50-50	Peak of B_1 with tailing
20mM Na_2HPO_4 pH 5.15 - MeOH	70-30	H_2O	t_{RB6}=4.5min - peak with tailing<br>t_{RB1}=7.5min - peak with tailing (Rs:4.85)<br>t_{RB2}=10.1min - sharp peak (Rs:3.37)
20mM Na2HPO4 pH 5.8 - MeOH	70-30	H_2O	t_{RB6}=4.5min - peak with tailing<br>t_{RB2}=10.1min - sharp peak<br>t_{RB1}=11.2min - peak with tailing (Rs:1.25)

20mM Na2HPO4 pH 7 - MeOH	70-30	H_2O	t_{RB6}=4.4min - peak with tailing<br>t_{RB2}=10.4min - sharp peak<br>t_{RB1}>15min
20mM Na_2HPO_4 pH 7 - MeOH	90-10	H_2O	t_{RB6}=7min - peak with tailing
20mM Na_2HPO_4 pH 7 - MeOH	95-5	H_2O	t_{RB6}=9min - peak with tailing

Stationary phase C18 Discovery HS (250 cm × 4.6 mm, 5 μm)
elution: isocratic, flow:0.9 ml/min, T: 40°C, injection volume: 30μl

Mobile phase	Mobile phase solvent ratios (%)	Diluent	Comments
20mM NaH_2PO_4 pH 4.5 - MeOH	70-30	H_2O	Peak of B_1 at the solvent front<br>Peak of B_6 after the solvent front<br>t_{RB2}=9.5min
20mM NaH_2PO_4 pH 5.15 - MeOH	70-30	H_2O	Peak of B_1 with tailing at the solvent front<br>Peak of B_6 after the solvent front<br>t_{RB2}=9.5min
20mM NaH_2PO_4 pH 5.8 - MeOH	70-30	H_2O	Peak of B_1 at the solvent front
20mM NaH_2PO_4 pH 6.8 - MeOH	70-30	H_2O	Peak of B_1 at the solvent front
20mM NaH_2PO_4 pH 6.8 - MeOH	90-10	H_2O	t_{RB1}=5min<br>t_{RB6}=6.1min- Rs<3<br>t_{RB2} >20min

Stationary phase C18 Discovery HS (250 cm × 4.6 mm, 5 μm)
elution: gradient, flow:0.7 ml/min, T: 40°C, injection volume: 30μl

Mobile phase	Mobile phase solvent ratios (%)	Diluent	Comments
20mM NaH_2PO_4 pH 4.5 - MeOH	90-10=> 60-40	H_2O	t_{RB1}=6.4min<br>t_{RB6}=7.7min<br>t_{RB2}=13.8min

Stationary phase Phenyl-ACE® (150 mm ×4.6 mm, 5 μm)
elution: isocratic, flow:1 ml/min, T: 25°C, injection volume: 30μl

Mobile phase	Mobile phase solvent ratios (%)	Diluent	Comments

20mM NaH_2PO_4 pH 6.7 - MeOH	90-10	H_2O	t_{RB1}=12min – peak with shoulder
20mM NaH_2PO_4 pH 6.7 - ACN	85-15	H_2O	t_{RB1}=6min - peak with shoulder
20mM NaH_2PO_4 pH 6.7 - ACN	80-20	H_2O	t_{RB6}=2.2min<br>t_{RB2}=2.9min - Rs:1.5<br>t_{RB1}=5.3min - peak with shoulder
20mM NaH_2PO_4 pH 6.7 - ACN	90-10	H_2O	t_{RB6}=2.4min<br>t_{RB2}=7.6min - peak with shoulder<br>t_{RB1}=8.6min - peak with shoulder (Rs<2.5)
**Stationary phase CN Waters Spherisorb® (250 mm×4.6 mm, 5 μm)<br>elution: isocratic, flow:1 ml/min, T: 25°C, injection volume: 30μl**			
**Mobile phase**	**Mobile phase solvent ratios (%)**	**Diluent**	**Comments**
20mM NaH_2PO_4 pH 3 - ACN	95-5	H_2O	t_{RB2}=6.2min<br>t_{RB6}=12.3min - peak with tailing<br>t_{RB1}=25.5min - peak with tailing

Table S3. Physicochemical properties of vitamins B_1, B_2 and B_6.

	**B_1**	**B_2**	**B_6**
Molecular Weight (g/mol)	265.36	376.4	169.18
Log P	1	-1.5	-0.8
pka (strongest acidic)	15.5	5.97	9.4
pka (strongest basic)	5.54	-2.6	5.58
Water Solubility (mg/ml)	500	0.1	200

Figure S1a. UV spectra of vitamins B1, B2 and B6.

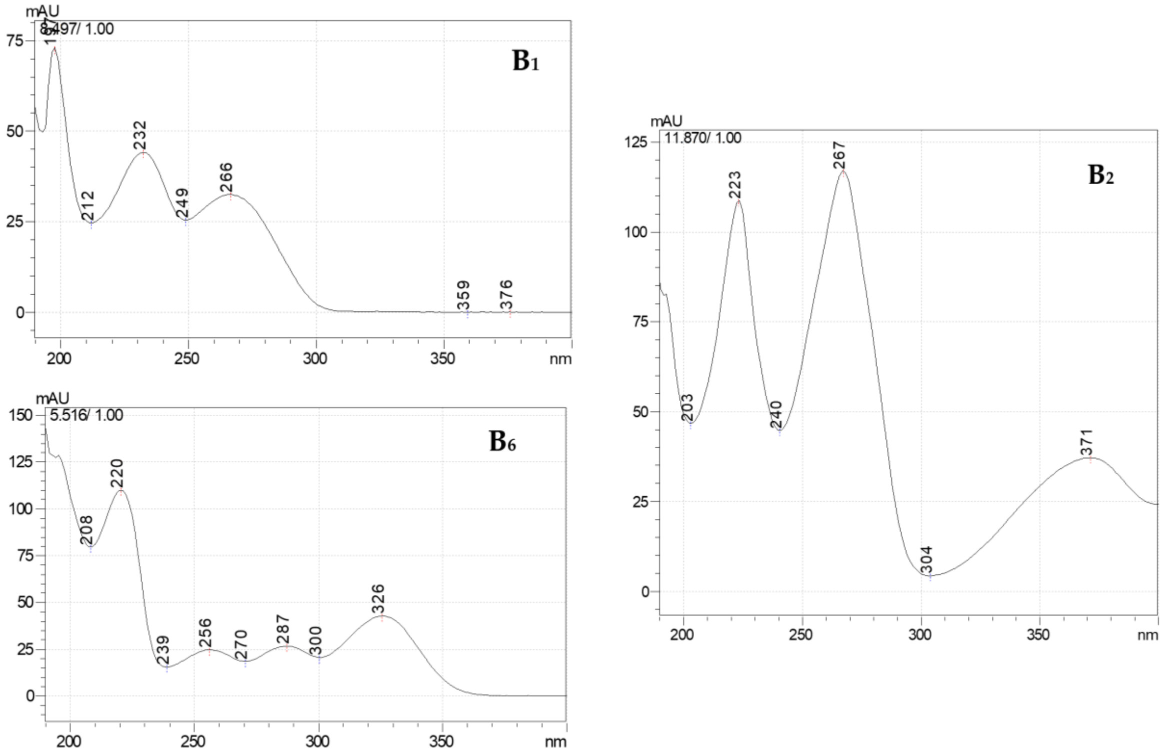

Figure S1b. FLD spectra of vitamins Thiochrome and Vitamins B2 and B6.

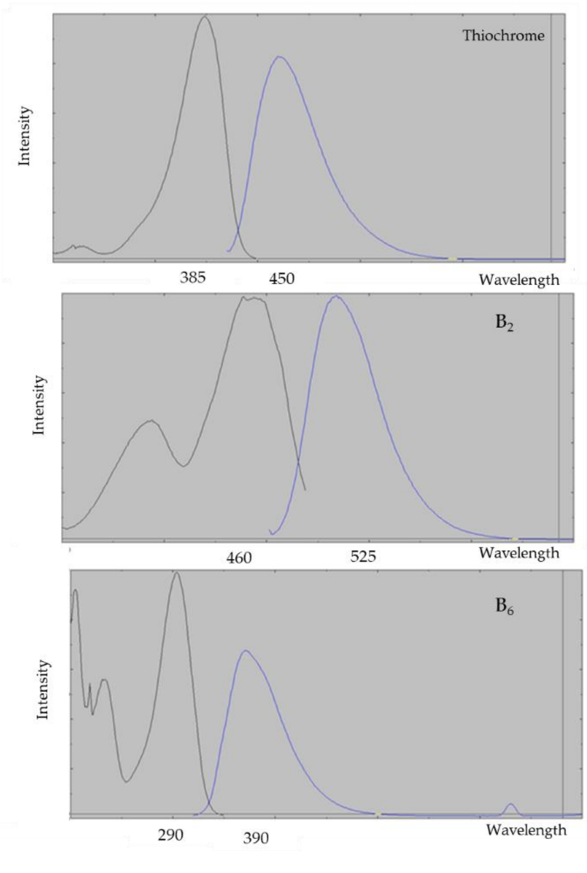

Table S4. Stability of B_1, B_2 and B_6 in 15 minutes after derivatization procedure at different temperatures

**Temperature (° C)**	**B_1 Stability (%)**	**B_2 Stability (%)**	**B_6 Stability (%)**
-18	75.2	100.4	100.1
4	69.6	100.2	100.2
25	51.8	99.9	99.8
70	28.6	51.8	99.3

Table S5. Accuracy test for both UV and FLD detectors.

	**HPLC-UV**					
Concentrations	B_1		B_2		B_6	
	Found	%Recovery	Found	%Recovery	Found	%Recovery
1.6/0.8/0.8 µg/mL	1.7	103	0.8	99.4	0.8	102
2.4/1.2/1.2 µg/mL	2.4	100.9	1.2	101.7	1.2	100
8/4/4 µg/mL	7.8	97.6	4	99.3	4	98.8
18/9/9 µg/mL	18.1	100.5	8.8	98.1	8.9	98.7
40/20/20 µg/mL	40.1	100.3	20	100.2	20	100.3
			**HPLC-FLD**			
60/4/4 ng/mL	60.3	100.6	4.1	101.8	3.9	98.3
200/20/20 ng/mL	205.4	102.7	19.7	98.3	20.4	102
400/40/40 ng/mL	410.1	102.5	39.2	98	39.9	99.7
800/80/80 ng/mL	823.3	102.9	80.2	100.3	81.03	101.3
1600/ 160/160 ng/mL	1607.8	100.5	161.7	101.1	163.1	101.9

Table S6. %Recoveries of methanolic solution of B_1, B_2, B_6, as a function of time, after ultrasound and heating.

	% Recovery		
min	B_1	B_2	B_6
0	100.0	100.0	100.0
15	100.16	94.37	100.7
30	99.43	94.47	99.55

Table S7. % Recovery of B_1, B_2 and B_6 from the gummy formulation.

sample	% Recovery		
	B_1	B_2	B_6
1	97.0	96.3	97.8
2	98.5	101.7	102.6
3	106.2	106.4	101.7
4	95.6	96.5	96.6
5	101.6	98.7	101.5
Mean	99.81	99.9	100.0
%RSD	4.22	2.18	2.66

Table S8. %Recoveries values in SGF and SIF samples after SPE, in presence of water, orange juice and milk.

Simulated Fluid + Coadministrated Fluid	% Recovery (%RSD)		
	B_1	B_2	B_6
SGF + water	102.9 (3.2)	99.3 (2.6)	103.2 (2.6)
SGF + juice	103.3 (3.5)	99.9 (4.1)	104.4 (3.1)
SGF + milk	104.1 (3.1)	99.8 (4.8)	107.1 (2.9)
SIF + water	99.6 (4.9)	109.5 (3.8)	95.3 (4.0)
SIF + juice	101.9 (3.1)	111.4 (4.6)	93.3 (4.8)
SIF + milk	101.5 (4.1)	112.8 (3.8)	96.0 (4.6)

Figure S2. Chemical structure of B_1, B_2, B_6.

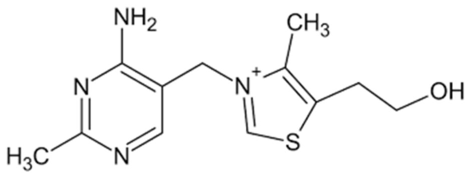

Thiamine (B1)

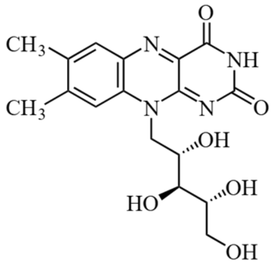

Riboflavin (B2)

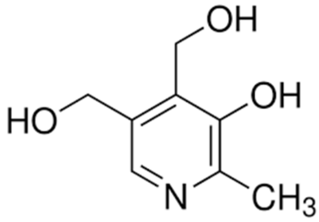

Pyridoxine (B6)

Text S1. Preparation of in vitro digestion fluids.

To prepare the SSF, a mixture of selected salts was used at specific final concentrations. The salts and their respective concentrations were as follows: KCl 15.1 mmol/L, KH_2PO_4 3.7 mmol/L, $NaHCO_3$ 13.6 mmol/L, $MgCl_2(H_2O)_6$ 0.15 mmol/L, $(NH_4)_2CO_3$ 0.06 mmol/L and $CaCl_2(H_2O)_2$ 1.5 mmol/L. The pH was adjusted to 7 using a 1 M HCl solution.

Similarly, for the SGF preparation, the salts were added to the following concentrations: KCl 6.9 mmol/L, KH_2PO_4 0.9 mmol/L, $NaHCO_3$ 25 mmol/L, NaCl 47.2 mmol/L, $MgCl_2(H_2O)_6$ 0.1 mmol/L, $(NH_4)_2CO_3$ 0.5 mmol/L and $CaCl_2(H_2O)_2$ 0.15 mmol/L. The pH was adjusted to 3 using a 1 M HCl solution.

Finally, for SIF, the final concentrations of the salts were: KCl 6.8 mmol/L, KH_2PO_4 0.8 mmol/L, $NaHCO_3$ 85 mmol/L, NaCl 38.4 mmol/L, $MgCl_2(H_2O)_6$ 0.33 mmol/L and $CaCl_2(H_2O)_2$ 8.4 mmol/L. The pH was adjusted to 7 using a 1 M HCl solution.

For the oral phase solution, 3.525 mL of SSF were added with 0.5 mL salivary α-amylase solution of 1500 U/mL (diluent SSF) and 975 μL of water.

For the gastric phase solution, the oral bolus was mixed with 7.505 mL of SGF, 1.6 mL porcine pepsin stock solution of 25 000 U/mL (diluent SGF), 0.2 mL of 1 M HCl and 695 μL of water.

For the intestinal phase solution, the gastric chyme was mixed with 11.04 mL of SIF, 5.0 mL of a pancreatin solution 800 U/mL (diluent SIF), 2.5 mL fresh bile (160 mM in fresh bile) and 1.31 mL of water. The final pH was adjusted at 7.

It is important to note that $CaCl_2$ $(H_2O)_2$ was added at the final stage—in the oral, gastric, and intestinal phase solutions—after pH adjustment and prior to the addition of water.
